# Antibody blockade of semaphorin 4D breaks down barriers to enhance tumoricidal immune infiltration and supports rational immunotherapy combinations

**DOI:** 10.1186/2051-1426-3-S2-P220

**Published:** 2015-11-04

**Authors:** Elizabeth E Evans, Siwen Hu-Lieskovan, Holm Bussler, Sebold Torno, Crystal Mallow, Christine Reilly, Maria Scrivens, Ekaterina Klimatcheva, Laurie A Winter, Renee Kirk, Alan Howell, Leslie Balch, Janaki Veeraraghavan, Alan S Jonason, John E Leonard, Mark Paris, Terrence L Fisher, Antoni Ribas, Ernest S Smith, Maurice Zauderer

**Affiliations:** 1 Vaccinex, Inc, Rochester, NY, USA; 2 David Geffen School of Medicine at UCLA, Los Angeles, CA, USA; 3 University of California at Los Angeles Medical Center, Los Angeles, CA, USA

## 

Semaphorin 4D (SEMA4D, CD100) and its receptor plexin-B1 are broadly expressed in cancer and expression correlates with invasive disease in several human tumors. SEMA4D normally functions to regulate the motility and differentiation of multiple cell types, including those of the immune, vascular, and nervous systems. In the setting of cancer, we describe a novel immunomodulatory function of SEMA4D in regulating immune cell infiltration and anti-tumor activity. Activity is enhanced in preclinical studies when combined with other immunotherapies, including immune checkpoint blockade inhibition.

## Methods

*In vitro* effects of SEMA4D and *in vivo* blockade of SEMA4D with monoclonal murine antibody was evaluated in preclinical syngeneic models – tumor growth and immune-mediated responses were characterized to evaluate mechanism of action. The safety and tolerability of humanized anti-SEMA4D antibody VX15/2503 was assessed in Phase I clinical trials in oncology.

## Results

SEMA4D restricts migration of macrophage cell lines *in vitro*. Strong expression of SEMA4D at the invasive margins of actively growing *in vivo* tumors modulates the infiltration and spatial distribution of leukocytes in the tumor microenvironment (TME). Antibody neutralization of SEMA4D disrupts this gradient and facilitates recruitment of activated antigen presenting cells and T lymphocytes into the TME, shifting the balance of cytokines toward increased Th1 and reduced immunosuppressive cytokines. This orchestrated change in the tumor architecture was associated with durable tumor rejection and immunologic memory in preclinical models. Importantly, the immunomodulatory activity of anti-SEMA4D antibody can be further enhanced by combination with other immunotherapies, including immune checkpoint inhibition and chemotherapy. Strikingly, the combination of anti-SEMA4D antibody with antibody to CTLA-4 acts synergistically to promote complete tumor rejection, with significant 58% increase in tumor regression and maximal increase in survival, as compared to monotherapy.

Treatment with anti-SEMA4D antibodies was well tolerated in nonclinical and clinical studies, including completion of a Phase I prospective multiple ascending dose trial in patients with advanced refractory solid tumors. Weekly doses of between 0.3 and 20 mg/kg were administered; no MTD was determined. Patients with the longest duration of treatment, 48-55 weeks, included colorectal, breast, and a papillary thyroid patient, who had a partial response by RECIST. Progression free survival correlated with elevated baseline lymphocyte counts, supporting an immune mediated mechanism of action for VX15/2503.

## Conclusion

Inhibition of SEMA4D represents a novel mechanism and therapeutic strategy to promote functional immune infiltration into the tumor and inhibit tumor progression. A Phase Ib/IIa trial of combination therapy with immune checkpoint inhibition is planned.

